# Assessing Caribbean Shallow and Mesophotic Reef Fish Communities Using Baited-Remote Underwater Video (BRUV) and Diver-Operated Video (DOV) Survey Techniques

**DOI:** 10.1371/journal.pone.0168235

**Published:** 2016-12-13

**Authors:** Dominic A. Andradi-Brown, Consuelo Macaya-Solis, Dan A. Exton, Erika Gress, Georgina Wright, Alex D. Rogers

**Affiliations:** 1 Department of Zoology, University of Oxford, The Tinbergen Building, South Parks Road, Oxford United Kingdom; 2 Operation Wallacea, Wallace House, Old Bolingbroke, Spilsby, Lincolnshire United Kingdom; 3 Departamento de Ciencias Ecológicas, Facultad de Ciencias, Universidad de Chile, Las Palmeras, Santiago, Chile; Academia Sinica, TAIWAN

## Abstract

Fish surveys form the backbone of reef monitoring and management initiatives throughout the tropics, and understanding patterns in biases between techniques is crucial if outputs are to address key objectives optimally. Often biases are not consistent across natural environmental gradients such as depth, leading to uncertainty in interpretation of results. Recently there has been much interest in mesophotic reefs (reefs from 30–150 m depth) as refuge habitats from fishing pressure, leading to many comparisons of reef fish communities over depth gradients. Here we compare fish communities using stereo-video footage recorded via baited remote underwater video (BRUV) and diver-operated video (DOV) systems on shallow and mesophotic reefs in the Mesoamerican Barrier Reef, Caribbean. We show inconsistent responses across families, species and trophic groups between methods across the depth gradient. Fish species and family richness were higher using BRUV at both depth ranges, suggesting that BRUV is more appropriate for recording all components of the fish community. Fish length distributions were not different between methods on shallow reefs, yet BRUV recorded more small fish on mesophotic reefs. However, DOV consistently recorded greater relative fish community biomass of herbivores, suggesting that studies focusing on herbivores should consider using DOV. Our results highlight the importance of considering what component of reef fish community researchers and managers are most interested in surveying when deciding which survey technique to use across natural gradients such as depth.

## Introduction

When conducting ecological monitoring programs it is important to select an appropriate sampling method. Within coral reef fish assessments it is well known that all sampling methods suffer from biases [1,2], yet it is often crucial to have accurate data on fish species abundance and biomass to inform management decisions. Therefore it is important to consider the appropriateness of different methods to assess fish populations based on individual target species, dominance of key trophic groups within the community, biogeographic region and locally-influenced fish behavioural adaptations [3–6].

Historically it was common to use destructive sampling to minimise bias when surveying reefs, with ichthyocides such as rotenone released over a small area of reef to allow collection of most individuals for identification [7]. In many cases, contemporary fish community assessments are informing conservation management or ecological research, making such destructive sampling techniques largely unethical and counterproductive. Using ichthyocides for sampling also makes long term monitoring challenging by their very nature. In place of destructive sampling, research ultimately moved to a reliance on underwater visual census (UVC) methods [8], with divers swimming transects and recording abundance and in some cases length estimates for fish within estimated transect boundaries. While cheap and easy to conduct, UVC has been extensively criticised for a lack of repeatability resulting from inconsistencies between observers [9,10], such as a large variation in transect boundary and visual fish length estimation [2]. These problems reduce statistical power for UVC surveys making it harder to separate genuine changes from observer differences, particularly in long-term monitoring with inevitable turnover in observers through time [10].

As a result there have been widespread calls to use video methods for fish community monitoring [11,12]. Videography allows observers to pause footage during analysis to consult fish identification guides or experts for help with identification. They also make monitoring programs involving multiple observers over several years easier to standardise, as identical videos can be used to train observers and control for biases. The development of stereo-video camera systems (SVS) facilitates length measurements, useful for estimating both fish lengths and transect boundaries [2], further reducing observer bias. Video is not without challenges though, as despite advances in underwater imaging systems, videos reduce clarity and present a restricted field of view compared to observers in the water [13].

Both in-water stereo diver-operated video (DOV) and stereo baited video camera drops (baited remote underwater video; BRUV) are widely used for reef fish surveys. Despite this, however, studies assessing differences between these contrasting SVS systems on tropical reefs are geographically limited to western Australia [4,14] and Fiji [6]. Patterns observed in the Indo-Pacific may not be true for tropical reefs in the western Atlantic, which has differing fish species richness, trophic structures and taxonomic groups providing ecosystem functions [15]. In addition, previous comparison studies have not considered differential effects of depth on the results obtained. Yet understanding any variation with depth between DOV and BRUV is crucial, as technique choice is often influenced by survey depth. Scientific divers conducting research within the recreational diving range are normally limited to 30 m maximum depth, with little scope to conduct in-water reef fish surveys deeper than 20 m because of breathing gas and no-decompression limit restrictions [16]. However, researchers are becoming increasingly interested in mesophotic reefs (light dependent reefs 30–160 m [17]) as potential refuges for fish from shallow-focused fishing pressure and other anthropogenic threats [18,19]. In addition, it is increasingly recognised that mesophotic reefs face many threats in their own right [20]. To survey deeper reefs many researchers use BRUV systems, because of the low cost and ease of surveying compared to the significant resources required for divers to safely work at mesophotic depths via technical diving [16]. On shallow reefs (<20 m) where monitoring programs historically have conducted UVC transects, DOV surveys are the logical video equivalent.

Many factors are known to affect fish detection in video surveys, and their impacts are likely to vary between BRUV and DOV, and also with depth. Between survey type, Watson and Harvey [21] found the presence of a diver in the water caused changes in the recorded abundance and approach distance for several fish species, while other species may be attracted to divers [22]. These biases are unlikely to be random with respect to fish trophic group, for example carnivorous snappers were under-detected by divers compared to remote camera surveys [23], and baiting camera systems may bias fish community surveys towards larger predatory fish [24]. This has lead to the suggestion that other trophic groups may appear at lower abundances on BRUVs, although bait has been shown to have little impact on recorded abundance of herbivores [25].

Many marine protected areas now have substantial recreational dive-based tourism on their shallow reefs. Reef fish have been shown to partially habituate to passive diver presence [26], suggesting differences between DOV surveys conducted on shallow heavily-dived reefs and deeper, less-frequently dived reefs may partially be caused by differing fish responses to divers. A recent study surveying protected and spear-fished areas found large differences in fish biomass detected by DOV, based on the diver equipment use [27]. Transects filmed using normal recreational open-circuit equipment recorded lower fish diversity and abundance when compared to near-silent closed-circuit rebreathers in the fished areas, suggesting fish evade detection in fished areas through diver avoidance. If deeper reefs are below the limit of fishers, this would not only lead to a biomass refuge [18,19], but also potentially make resident fish less evasive of diver surveys when compared to their shallow counterparts. In addition, many reef fish species exhibit ontogenetic migrations with new fish recruits settling in shallow marine habitats (i.e. mangroves, seagrass beds and shallow coral reefs) and moving to deeper reefs as they mature [28]. Maturation in many fish species is associated with changes in diet [29], suggesting individual fish species responses to bait could be dependent on individual fish maturity, which is correlated with depth. Maturation is also associated with changes in behaviour, as fish try to minimise predation risk while maximising feeding [30] and so potentially affecting detection ability by divers across the shallow to mesophotic reef gradient.

To test these questions, we compare BRUV and DOV assessments of fish community structure on shallow and mesophotic reefs in the western Atlantic, and assess whether differences between techniques are consistent across the depth range. Specifically, we test whether the baited nature of BRUV leads to a greater proportion of carnivorous fish in the community compared to DOV and contrast this with herbivorous reef fish detection differences between techniques and depths.

## Methods

### Study sites

**Fig 1:**
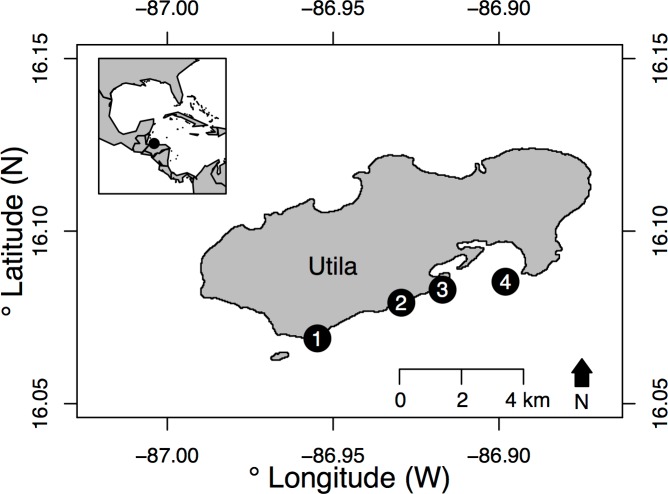
The four survey sites on the south shore of Utila, Bay Islands, Honduras. Sites were: (1) Stingray Point, (2) Little Bight, (3) Black Coral Wall and (4) Lighthouse Reef. See S1 Table for full GPS location data. Inset–The location of Utila is indicated with a black circle relative to the western Caribbean and Gulf of Mexico. Map sourced from GADM database of Global Administrative Areas (2015) under a CC BY license, used with permission.

Study sites were located on Utila, Honduras, approximately 29 km north of the Honduran mainland (Fig 1). Utila is at the southern extent of the Mesoamerican Barrier Reef, and extensive coral reefs and mangrove forests exist around the island. The reefs of Utila are contained within the Bay Islands National Marine Park, and there is a large recreational dive tourism industry [31], with tens of thousands of dives conducted on the shallow reefs annually. Despite this, there is a large fishery around the island [32,33], although increasingly this fishery focuses on offshore rather than fringing reefs, and so fishing is only conducted at low levels by hand lines at our study sites. Surveys were conducted at four sites on the south shore of Utila (Fig 1), where the shallow reef slopes down to the continental shelf (approximately 60–80 m depth), which extends to the Honduran mainland. All surveys were conducted in June-August 2014, with research permits issued by Instituto de Conservación Forestal (ICF), Honduras.

### Survey techniques

Surveys made use of stereo-video systems (SVS). SVS consist of two video cameras at fixed angles to allow footage of fish to be filmed simultaneously from two different positions. Using these dual images it is possible to accurately estimate the length of fish. These lengths can be converted into biomass estimates using standardised length-weight relationships.

#### Diver-operated video (DOV)

Transects were conducted using a commercially available stereo-DOV (SeaGIS, Melbourne, Australia), fitted with two Cannon HFS21 cameras (see Watson et al. [4] for a full system overview). To minimise disturbance to the recorded fish community while setting up the cameras, transects were conducted as follows: the DOV operator started the cameras recording, synchronised them using a handheld torch and then angled the cameras downwards while their dive buddy attached a transect tape to the reef. The team then swam 10 m along the reef with the cameras pointing downwards as the buddy laid the transect tape, after 10 m the buddy signalled to the DOV operator. The DOV operator then started the 50 m transect by angling the cameras to film across the reef slope at the target survey depth. The DOV operator then swam along the reef with their buddy laying the transect tape and signalling when 50 m had been covered. Transects took approximately three minutes to film, with divers using open-circuit dive equipment. Four 50 m long transects were conducted at both 5 m (shallow) and 35–40 m (mesophotic) at each site, with each transect separated by a 10 m interval and following the respective depth contour (see S1 Table for GPS locations).

#### Baited remote underwater video (BRUV)

We used a custom made BRUV consisting of two GoPro Hero 3+ Silver Cameras mounted in ScoutPro H3 deep-sea housings on an aluminium bar 0.8 m apart and inwardly converging at 10 degrees. This was built into a plastic chassis with a weight. The BRUV was deployed for 50 minutes in two depth bands; 5 m (shallow) and 30–55 m (mesophotic). Five BRUV drops were conducted at each depth at each site, with the shallow BRUVs placed along the same reef crest location as the shallow DOV transects were conducted (see S1 Table for more details). The shallow BRUV deployments were assisted by divers to minimise damage to the reef and were placed at approximately 20 m intervals along the reef crest facing outwards towards open water. Mesophotic BRUVs were deployed by boat over the location of the mesophotic DOV transects, with a depth sounder used to find the target depth of 40 m prior to deployment, though actual depths recorded by the BRUV varied between 30–55 m (see S1 Table for BRUV GPS locations and depths). Each BRUV was baited with approximately 1.5 Kg of tuna heads and guts, suspended in a wire mesh bag 80 cm in front of the cameras. Tuna parts used were sourced from a local fisher, and were waste material that would normally be disposed of following filleting. Only one BRUV was deployed at a time, and multiple BRUV deployments at the same site were separated by a minimum of 2 hours to avoid overlaps of bait plumes.

The ordering of DOV transects and BRUV drops at each depth at each site was randomly selected, with no more than two BRUV drops or two DOV transects conducted in a depth band at a site on one day.

#### SVS analysis

Both BRUV and DOV camera systems were calibrated using a calibration cube and CAL software (http://www.seagis.com.au/bundle.html). All fish length measurements were conducted in EventMeasure software (http://www.seagis.com.au/event.html). Fish lengths were measured as fork lengths, the distance from the tip of the snout to end of the centre of the caudal fin rays.

For BRUVs, the MaxN biomass for each species was recorded. Videos were watched for 50 minutes from the BRUV camera system arriving on the seabed, and video frames were annotated in EventMeasure to indicate the paired frames containing the maximum number of individuals of each species during this period. The length of all the fish in this frame was then measured. MaxN avoids repeatedly counting the same individual, as fish often enter, exit and then re-enter the view for static cameras [34].

To ensure consistency within the DOV analysis, only fish that had their mid-point within a 5 m transect width (2.5 m either side of the camera centre) were included, and only fish within 5 m linearly in-front of the cameras were included. Fish with a three-dimensional location outside these specifications when calculated in EventMeasure were ignored, enabling us to standardise the DOV survey area. This generated a total survey area of 250m^2^ of reef per DOV transect. Care was taken to watch fish swimming behaviour on transects to minimise risk of double counting individuals that moved along the reef as we swam. Because of the relatively short survey time of the DOV transect (3 min), and the linear distance covered along the reef while surveying (50 m), the risk of double counting fish was low. The total abundance of each fish species and all their individual lengths across the whole transect were then used to estimate the fish community.

For both BRUV and DOV it was sometimes not possible to measure the length of a fish as it appeared on one camera only of the stereo pair, normally caused by being close in front of one of the cameras. In these cases, the mean length of all other individuals of that species recorded on that BRUV drop or DOV transect was applied. If no other individuals of the species were recorded on that drop or transect, the mean of all other individuals of the species across all BRUV drops or DOV transects at that site and depth band were used. All lengths were converted into weight estimates using Eq 1, where *W* is the fish weight (g), *L* is the fish length (cm) and *a* and *b* species-specific conversion constants.

$$W=aL^{b}$$ Equation 1

Conversion constants were obtained from fishbase (accessed September 2014 [35]). Full raw data is provided in S1 Data.

### Data analysis

Fish species were allocated into three trophic groups based on the feeding guild classification by Micheli *et al* [36]. Trophic groups were: herbivores, carnivores (grouping ‘piscivores’ and ‘invert. feeder/pisciv.’ from the classification) and others. Permutational multivariate analyses of variance (permutational MANOVA [37]) were used because of their lack of assumptions about data distributions. To test for differences in species richness, abundance, total biomass and relative biomass between trophic groups we used Euclidian distances in a permutational analysis of variance (permutational ANOVA). As DOV records all individual fish along transects, while BRUV records the MaxN of each fish species in a fixed location, comparing the raw recorded fish abundance or biomass between the techniques makes interpretation of patterns difficult. As our focus was on broader fish community differences between methods across depths we standardised fish abundance and biomass data by calculating relative abundance and biomass for each fish family and species on each transect or drop. This was done by dividing the total abundance or biomass of each fish family or species in turn by the total fish abundance or biomass recorded on the transect or drop. For community analysis, where many species were recorded as zero abundance and biomass we used Bray-Curtis dissimilarities on relative abundance or biomass made up by each species. Permutational ANOVA/MANOVA [37] was conducted using the ‘adonis’ function, while principle coordinates analysis (PCO) was conducted using the ‘cmdscale’ function, both from the package vegan [38] in R [39]. All permutational tests were run for 9999 permutations, constrained within survey site, and simplified to remove non-significant interactions between model terms. As our study had multiple factors and unbalanced numbers of samples between the two survey methods, when testing for interactions the sums of squares for model terms are non-independent [37]. We used Type I (sequential) sums of squares, where each term was fitted sequentially after the previous fitted terms, meaning the order terms were fitted was important. This approach is appropriate when there is a logical order to fit terms based on the research question being addressed [37], in this study we wanted to identify the effect of depth after the effect of site, and the effect of survey method after the effects of site and depth. We therefore fitted terms in the following prioritisation order: site, depth and survey method, with interactions in the order: site:depth, site:method, depth:method, site:depth:method.

A constrained analysis of principal coordinates (CAP) was run for all shallow and mesophotic data separately using the function ‘capscale’ in vegan [38]. We tested for fish species likely to be driving differences between the two methods and depths by identifying Pearson correlations between individual fish species’ relative community biomass and the canonical axis. Correlations of |*r*|≥0.3 were used to highlight potential species which might be driving differences. We also plotted relative community biomass for each species assessed by BRUV against that assessed by DOV, allowing species showing large differences in community composition between the two methods to be identified.

Kernel density estimates (KDE) were used to calculate length distributions for fish recorded by each method. KDEs were calculated using the ‘dpik’ function in the package KernSmooth [40]. The ‘dpik’ function selects KDE bandwidths using the Sheather-Jones selection procedure, which selects the optimal bandwidth for constructing a KDE based on the distribution of the lengths [41]. We followed Langlois *et al*. [42] in using the function ‘sm.density.compare’ from the R package sm [43] to test whether KDEs generated for fish communities surveyed by the two methods in each depth band were significantly different.

## Results

### Species richness, abundance and biomass

**Fig 2:**
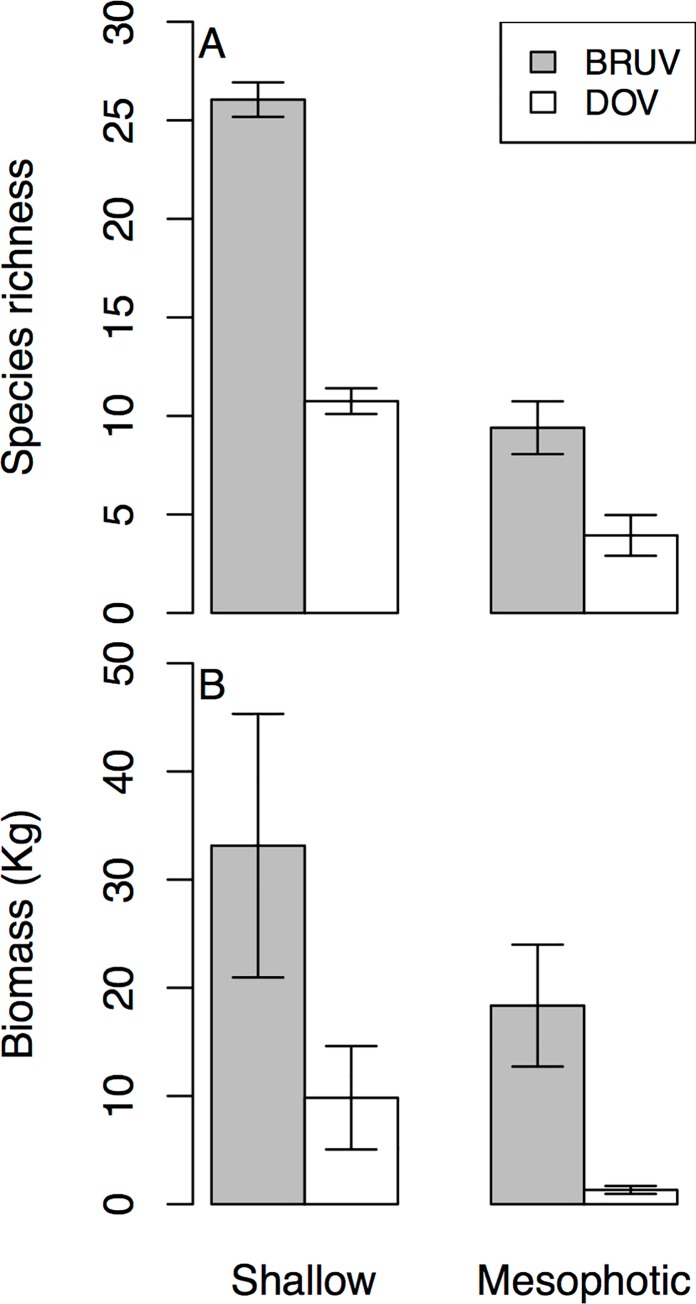
**Mean (A) fish species richness and (B) fish biomass of shallow and mesophotic reefs per site using the two different sampling methods (BRUV and DOV).** Species richness and biomass is per 250m^2^ transect for DOV, and per BRUV drop for BRUV data.

**Fig 3:**
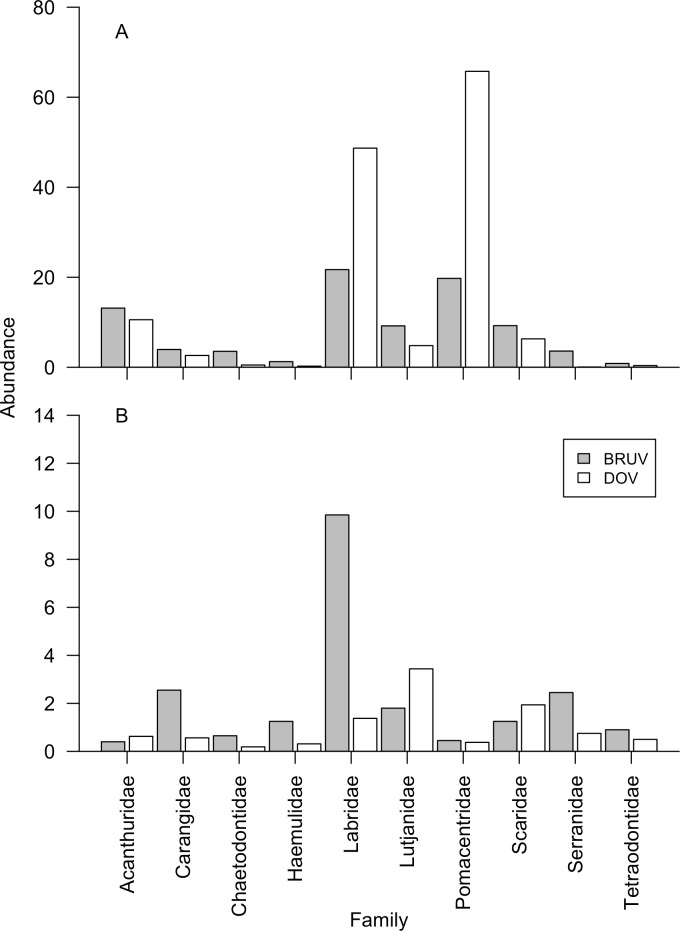
**Fish abundance per family on (A) shallow reefs, and (B) mesophotic reefs, using BRUV and DOV methods calculated across all sites.** Only families comprising >1% of the total number of fish recorded are shown. Abundance represents the mean number of fish recorded per 250m^2^ DOV transect, or per BRUV drop for DOV and BRUV surveys respectively.

On shallow reefs DOV recorded 26% more individual fish than BRUV, while on mesophotic reefs BRUV recorded 339% more individuals than DOV (Table 1). For both shallow and mesophotic reefs BRUV recorded more species than DOV, with 72% and 88% respectively, and many species unique to each method (Fig 2A). BRUV at both shallow and mesophotic depths also recorded more families than DOV. The top five most commonly recorded fish families are identical for shallow reefs between the two methods (Labridae, Pomacentridae, Acanthuridae, Scaridae and Lutjanidae), but differ for mesophotic reefs (Fig 3). For mesophotic reefs both techniques recorded Labridae, Serranidae and Lutjanidae in their top five families, but BRUVs also recorded Carangidae and Sparidae while DOV recorded Scaridae and Acanthuridae. We tested for differences in species richness between sites, depths and methods (S2 Table), finding that there were significant site:method interactions and depth:method interactions, suggesting that identified differences in fish species richness between sites is affected by survey method, but more importantly that the difference in species richness recorded by BRUV and DOV is affected by depth. Mean species richness was greatest on shallows reefs for both methods, declining 64% for BRUV and 63% for DOV when compared to mesophotic reefs (Fig 2). We found fish biomass was significantly affected by site, depth and the survey method, with a significant site:depth interaction, suggesting that fish biomass changes differently across the depth gradient depending on site (S3 Table). Mean fish biomass recorded per replicate was greater for BRUVs than DOV at both depths (Fig 2B), but declined with depth.

**Table 1:** Summary of collected fish data on shallow and mesophotic reefs by diver-operated (DOV) and baited remote (BRUV) stereo-video systems.

	Shallow reefs			Mesophotic reefs		
	BRUV	DOV	Total	BRUV	DOV	Total
Individuals	1792	2251	4043	514	117	631
Species	74	43	80	60	32	69
Species unique to method	37	6		37	9	
Families	22	15	23	25	16	26
Families unique to method	8	1		10	1	

### Fish community structure

For fish families on shallow reefs, DOV recorded a greater mean abundance of Labridae and Pomacentridae per transect than BRUV recorded per drop (Fig 3A). This is reflected in greater relative community abundances for Labridae and Pomacentridae on DOV than BRUV at 35% and 47% versus 24% and 22% respectively. BRUV recorded greater abundances per drop than per DOV transects for Chaetodontidae and Serranidae on shallow reefs, with other fish families appearing at similar abundances from both methods (Fig 3A). On mesophotic reefs the pattern was reversed for Labridae, with BRUV recording greater abundance than DOV (Fig 3B), and Labridae comprising 38% of the community by abundance. Herbivorous families such as Acanthuridae and Scaridae were recorded at similar abundances by BRUV and DOV on both shallow and mesophotic reefs (Fig 3). Interestingly, the carnivorous family Lutjanidae was recorded at higher abundances on mesophotic reefs by DOV than BRUV. While Carangidae, another carnivorous family, showed the reverse of this pattern with greater BRUV abundance than DOV on mesophotic reefs.

**Fig 4:**
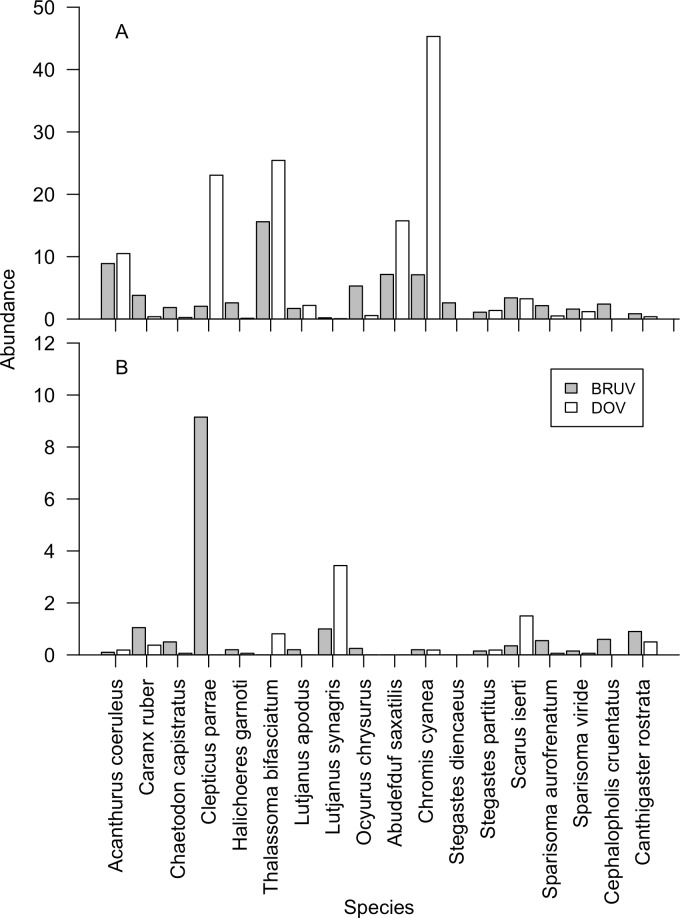
**Fish abundance per species on (A) shallow reefs, and (B) mesophotic reefs, using BRUV and DOV methods calculated across all sites.** Only species comprising >1% of the total number of fish recorded are shown. Abundance represents the mean number of fish recorded per 250m^2^ DOV transect, or per BRUV drop for DOV and BRUV surveys respectively.

At the species level, several different species dominate the communities at shallow and mesophotic depths (Fig 4), driving the previously reported method:depth interactions. The most common shallow water fish recorded by DOV was the damsel *Chromis cyanea*, making up 32% of the fish community compared to only 8% on BRUV (Fig 4). *Clepticus parrae* was also recorded at greater relative abundance on DOV (16%) than BRUV (2%) on shallow reefs. At mesophotic depths, however, *C*. *parrae* was the most abundant fish species recorded by BRUV, composing 37% of the fish community. Other notably abundant species at mesophotic depths are the snapper *Lutjanus synagris* and the parrotfish *Scarus iseri*, both of which were recorded at high abundances by DOV.

We calculated relative biomass of each fish species recorded to investigate patterns in fish community structure. When visualising the relative community biomass of each species using PCO, at most sites the communities grouped primarily based on depth, with some grouping based on method within depth bands (S1 Fig). When formally tested we found differences in fish community structure between different sites, depths and methods and also site:method and depth:method interactions (S4 Table). We conducted CAP analyses to identify families and species driving this pattern. On shallow reefs our CAP analysis indicated BRUVs tended to be correlated with greater relative biomasses of families that contain carnivores such as Haemulidae, Lutjanidae and Serranidae, though also surprisingly Chaetodontidae (Table 2). For both shallow and deep reefs we found that DOV detected a greater relative community biomass of Scaridae. On deep reefs BRUV detected Dasyatidae, Ostraciidae and Pomacanthidae that were absent from DOV surveys. Unsurprisingly many species correlating with our species level CAP axis (Table 3) belonged to families correlated with our family level CAP axis (Table 2). Five species of Pomacentridae (*Abudefduf saxatilis*, *Chromis cyanea*, *Chromis multilineata*, *Stegastes adustus* and *Stegastes partitus*) and three species of Scaridae (*Scarus coeruleus*, *Scarus iserti* and *Sparisoma viride*) made up a larger proportion of the shallow DOV biomass weighted community than BRUV. We found the carnivores *Lutjanus analis*, *Lutjanus jocu*, *Cephalopholis cruentatus* and *Mycteroperca venenosa* all as a greater proportion of the community biomass on shallow reef BRUV, alongside two Labridae species: *Bodianus rufus* and *Halichoeres radiatus*. One Labridae species, *Thalassoma bifasciatum*, was recorded as a greater relative biomass on DOV on both shallow and mesophotic reefs. At mesophotic depths we found five species (*Dasyatis americana*, *Haemulon aurolineatum*, *Gymnothorax moringa*, *Pomacanthus arcuatus* and *Cephalopholis cruentatus*) from five different families making up a greater proportion of relative community biomass on BRUV than DOV. With the exception of *Pomacanthus arcuatus* (a spongivore) these species are all carnivorous.

**Table 2:** Fish families with relative community biomass correlating (|r|≥0.3) with the constrained analysis of principle coordinates axis.

	*r*	BRUV		DOV	
		Mean±SE	Median	Mean±SE	Median
**Shallow**					
*BRUV > DOV*					
Chaetodontidae	-0.31	0.01 ± 0.01	0.01	0.01 ± 0.01	0.01
Haemulidae	-0.39	0.04 ± 0.01	0.05	0.04 ± 0.02	0.03
Lutjanidae	-0.52	0.25 ± 0.04	0.27	0.14 ± 0.03	0.13
Serranidae	-0.45	0.04 ± 0.01	0.04	0 ± 0	0
*DOV > BRUV*					
Pomacentridae	0.69	0.06 ± 0.02	0.05	0.24 ± 0.11	0.16
Scaridae	0.43	0.15 ± 0.04	0.13	0.24 ± 0.07	0.27
**Deep**					
*BRUV > DOV*					
Dasyatidae	-0.34	0.59 ± 0.01	0.59	0 ± 0	0
Ostraciidae	-0.30	0.07 ± 0.01	0.07	0 ± 0	0
Pomacanthidae	-0.31	0.12 ± 0.05	0.14	0 ± 0	0
Sparidae	-0.53	0.19 ± 0.05	0.16	0.08 ± 0	0.08
*DOV > BRUV*					
Labridae	0.31	0.02 ± 0.01	0.00	0.12 ± 0.07	0.10
Scaridae	0.40	0.03 ± 0	0.03	0.16 ± 0.04	0.12
Tetraodontidae	0.35	0 ± 0	0.00	0.13 ± 0.07	0.13

Fish families with relative community biomass correlating (|*r*|≥0.3) with the constrained analysis of principle coordinates axis indicating greater relative community biomass recorded for the fish family on one survey method over the other (DOV vs BRUV). Mean (± 1 standard error) and the median relative community biomass recorded for each family by both methods are reported.

**Table 3:** Fish species with relative community biomass correlating (|*r*|≥0.3) with the constrained analysis of principle coordinates axis.

	Species	*r*	BRUV		DOV	
			Mean±SE	Median	Mean±SE	Median
**Shallow**						
*BRUV > DOV*						
Acanthuridae	*Acanthurus chirurgus*	-0.36	0.01 ± 0	0.01	0 ± 0	0
Chaetodontidae	*Chaetodon striatus*	-0.40	0 ± 0	0	0 ± 0	0
Labridae	*Bodianus rufus*	-0.32	0.01 ± 0	0.01	0.01 ± 0.01	0
Labridae	*Halichoeres radiatus*	-0.32	0 ± 0	0	0 ± 0	0
Lutjanidae	*Lutjanus analis*	-0.35	0.07 ± 0.03	0.04	0.01 ± 0.01	0
Lutjanidae	*Lutjanus jocu*	-0.30	0.1 ± 0.02	0.11	0.08 ± 0.05	0.08
Pomacentridae	*Stegastes diencaeus*	-0.60	0 ± 0	0	0 ± 0	0
Serranidae	*Cephalopholis cruentatus*	-0.55	0.04 ± 0.01	0.04	0 ± 0	0
Serranidae	*Mycteroperca venenosa*	-0.34	0.01 ± 0	0.01	0 ± 0	0
*DOV > BRUV*						
Labridae	*Thalassoma bifasciatum*	0.44	0.01 ± 0	0.01	0.02 ± 0	0.02
Pomacentridae	*Abudefduf saxatilis*	0.37	0.03 ± 0.01	0.02	0.11 ± 0.08	0.04
Pomacentridae	*Chromis cyanea*	0.74	0 ± 0	0	0.12 ± 0.04	0.11
Pomacentridae	*Chromis multilineata*	0.49	0 ± 0	0	0.01 ± 0	0.01
Pomacentridae	*Stegastes adustus*	0.62	0 ± 0	0	0 ± 0	0.01
Pomacentridae	*Stegastes partitus*	0.40	0 ± 0	0	0 ± 0	0
Scaridae	*Scarus coeruleus*	0.31	0 ± 0	0	0.01 ± 0	0.01
Scaridae	*Scarus iserti*	0.37	0.01 ± 0	0.01	0.06 ± 0.02	0.05
Scaridae	*Sparisoma viride*	0.49	0.02 ± 0	0.02	0.12 ± 0.05	0.12
**Deep**						
*BRUV > DOV*						
Dasyatidae	*Dasyatis americana*	-0.47	0.59 ± 0.59	0.01	0 ± 0	0
Haemulidae	*Haemulon aurolineatum*	-0.32	0.01 ± 0.01	0	0 ± 0	0
Muraenidae	*Gymnothorax moringa*	-0.31	0.02 ± 0	0.01	0 ± 0	0
Pomacanthidae	*Pomacanthus arcuatus*	-0.32	0.2 ± 0.2	0.01	0 ± 0	0
Serranidae	*Cephalopholis cruentatus*	-0.4	0.01 ± 0.01	0	0 ± 0	0
*DOV > BRUV*						
Labridae	*Thalassoma bifasciatum*	0.30	0 ± 0	0	0.11 ± 0.07	0.10
Scaridae	*Scarus iserti*	0.51	0.01 ± 0	0	0.12 ± 0.02	0.12
Tetraodontidae	*Canthigaster rostrata*	0.31	0 ± 0	0	0.13 ± 0.07	0.13

Fish species with relative community biomass correlating (|*r*|≥0.3) with the constrained analysis of principle coordinates axis, indicating greater relative community biomass recorded for the fish species on one survey method over the other (DOV vs BRUV). Mean (± 1 standard error) and the median relative community biomass recorded for each species by both methods are reported.

**Fig 5:**
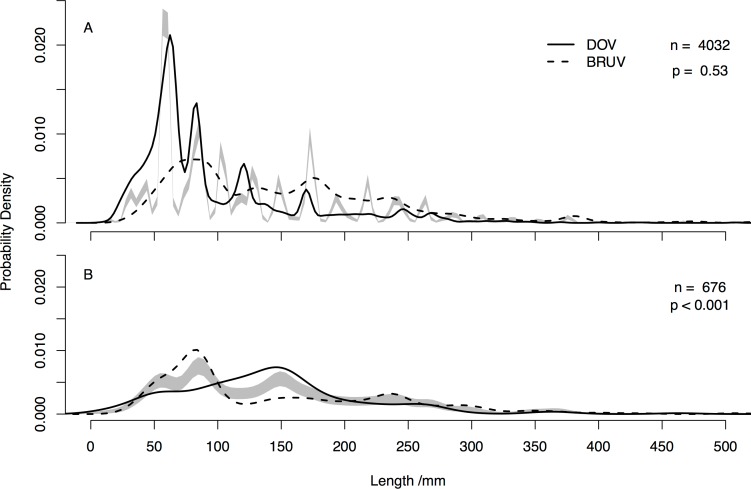
**Fish length frequency distribution for (A) shallow and (B) mesophotic reefs recorded by DOV and BRUV across all sites.** Grey shaded areas represent one standard error either side of the null model, *n* = number of individual fish measured, *p* indicates whether the length distributions are significantly different based on permutation tests.

We compared overall fish length distributions recorded by the two techniques on shallow and mesophotic reefs. On shallow reefs there was no difference between BRUV and DOV (Fig 5A), though DOV surveys generated several peaks, with many small fish. For mesophotic reefs there were more small fish recorded by BRUV than DOV surveys (Fig 5B).

### Herbivores and carnivores

As it has previously been suggested that using bait may bias recorded fish communities towards carnivores at the expense of other trophic groups, we conducted an analysis of just carnivores and herbivores. For both groups greater relative biomass was detected by DOV at mesophotic depths than BRUV (S2 Fig, S5 Table). Interestingly, there was no difference in relative biomass between techniques on shallow reefs for carnivores but DOV recorded higher relative biomass for herbivores (S2 Fig).

**Fig 6:**
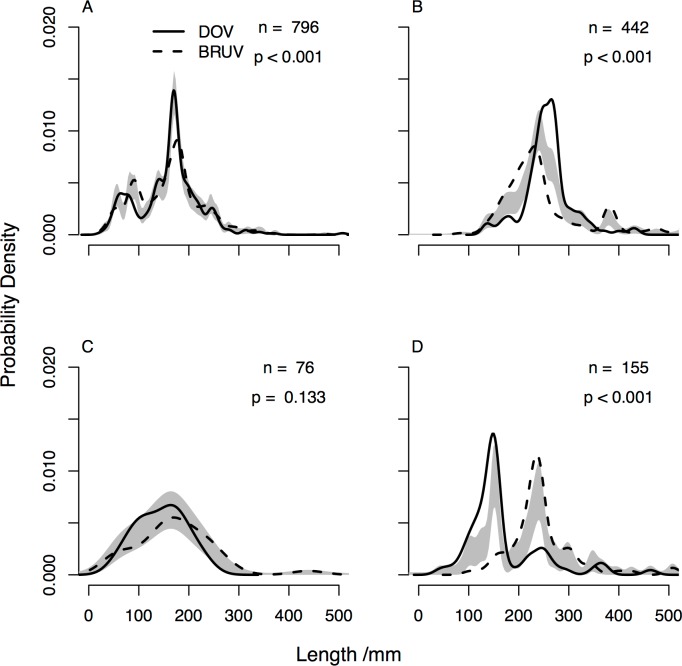
**Length distributions for shallow (A) herbivores, (B) carnivores and mesophotic (C) herbivores and (D) carnivores comparing BRUV and DOV.** Grey shaded areas represent one standard error either side of the null model, *n* = number of individual fish measured, *p* indicates whether the length distributions are significantly different based on permutation tests.

On shallow reefs we found differences in the herbivore length distribution (Fig 6A), with DOV generating a more distinctive modal peak, while on mesophotic reefs no difference was detected (Fig 6C). For carnivores differences in fish length distribution were detected at both shallow and mesophotic depths. On shallow reefs the mode was shifted to the right for DOV (Fig 6B), indicating larger carnivorous fish were more commonly detected when surveying by DOV, yet on mesophotic reefs the reverse was true, with larger carnivorous fish more commonly recorded on the BRUV (Fig 6D).

**Fig 7:**
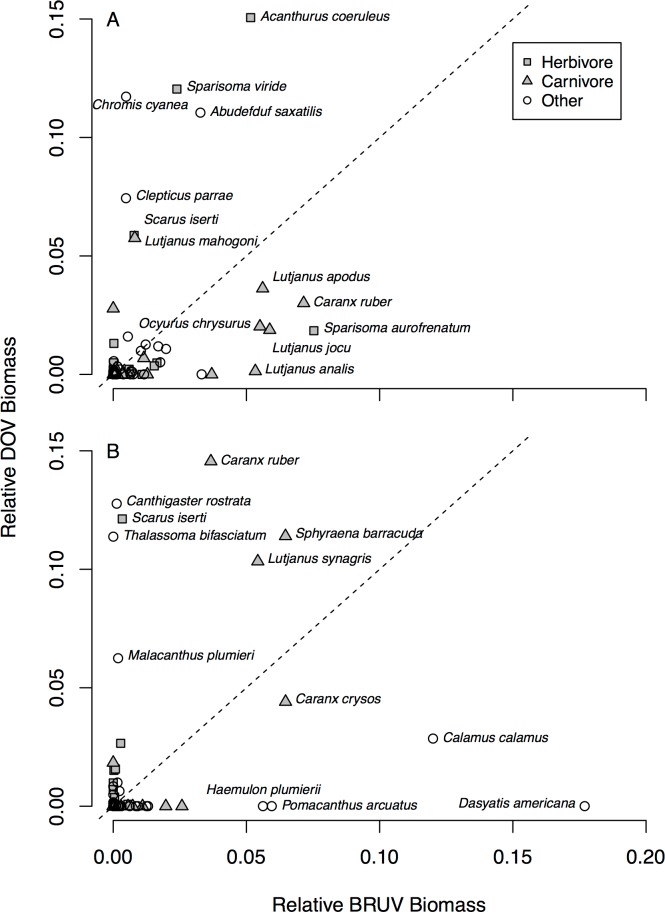
**Relative community biomass measured by BRUV compared to DOV for (A) shallow and (B) mesophotic reefs.** Species composing the same proportion of the community when assessed by both methods should be located on the dashed line, with species furthest from the dashed line showing significant bias towards one survey method.

To further identify which herbivore and carnivore species may be driving differences between the two survey techniques we plotted relative biomass surveyed by BRUV against that from the DOV (Fig 7). Fig 7A shows a group of carnivorous fish (*Lutjanus spp*., *Ocyurus chrysurus* and *Caranx ruber*), which were all recorded at greater proportions of the community on BRUV than DOV on shallow reefs. Herbivore species show less clear patterns in the shallows. At mesophotic depths the patterns are less clear (Fig 7B), though several carnivores appear to make up a larger proportion of the community on DOV than BRUV (*Caranx ruber*, *Sphyraena barracuda* and *Lutjanus synagris*).

## Discussion

In this study we compared diver-operated stereo-video surveys (DOV) and baited remote underwater video surveys (BRUV) on shallow and mesophotic coral reefs to identify differences in relative community biomass for fish species. Our results show the choice of sampling method affects the reef fish community results obtained, but crucially that these effects are not consistent with depth. This suggests fish families and species show differences in response to, or detection by, specific survey techniques depending on depth. Our results are particularly important for researchers assessing patterns in fish communities across depth gradients.

There are several possible explanations for inconsistent differences between BRUV and DOV surveys with depth, including differing fish responses to divers based on (i) historical diver exposure [26], (ii) fishing pressures [27] and (iii) ontogenetic changes with depth [28]. This study was conducted in the Bay Islands National Marine Park, though the sites we surveyed are not no-take zones. In practice, there is a large recreational dive tourism industry on Utila and multiple dive boats visit these sites in a typical day. This means fishing only occurs at low levels at the sites, but exposes the shallow reef fish community to intensive diver contact. Historically, however, fishing has been extensively conducted on the reefs of Utila [32,33].

Regardless of depth, and in line with other studies [4,14], we recorded more species on BRUV surveys than DOV. DOV recorded many more individual fish on shallow reefs than BRUV, reflecting the difference in counting fish between the two methods, with all fish counted on DOV transects, but only the maximum number of individuals seen in a single frame (MaxN) for BRUV [34]. Surprisingly, this pattern reversed on mesophotic reefs, with more individuals counted on BRUV than DOV. Species richness and abundance generally increases with increased sampling effort, therefore these patterns are likely to be influenced by our choice of BRUV drop time and size of DOV transect. However, fish abundances are known to be lower on mesophotic reefs than shallow reefs around Utila [44]. Therefore, greater fish abundance recorded by BRUVs on mesophotic reefs than DOVs might be caused by: the baited nature of BRUV, combined with the longer survey duration, allowing fish to be attracted from a larger area than that covered by DOV transects, or that BRUVs are better at detecting individual fish on mesophotic reefs. While it is possible to estimate the visible distance in a BRUV frame [45], this is not a good estimate of the survey area for many fish species, as bait plumes will spread varying distances and directions based on tides and currents. Studies looking at bait dispersal have found differences in fish community composition based on water flow rates past the BRUV [46]. Without better modelling of this, it is difficult to estimate bait plume area, especially with the BRUV left in place for 50 minutes.

On shallow reefs we found many carnivorous families (e.g. Lutjanidae and Serranidae) and species (e.g. *Lutjanus analis*, *Lutjanus jocu*, *Cephalopholis cruentatus and Mycteroperca venenosa*) significantly correlated with our CAP analysis indicating greater relative community biomass on BRUV than DOV. When looking at outlying species with greater relative biomass on BRUV than DOV on shallow reefs (Fig 7A) many *Lutjanus* species were identified alongside *Ocyurus chrysurus* and *Caranx ruber*. This increased biomass of carnivorous fish on BRUV fits with our hypothesis and with previous studies [14,25]. This is likely to be caused by a range of factors, including bait attraction [25], large bodied carnivorous species avoiding the bubbles produced by recreational divers particularly in areas with previous spearfishing [27], and the long BRUV deployment times [46,47].

Surprisingly we found greater carnivore biomass on mesophotic reefs on DOV than BRUV, but no particular carnivorous families or species correlated with our CAP analysis making it harder to identify specific carnivorous families or species driving this trend. However, three carnivorous species had greater relative biomass on DOV surveys than BRUV; *Caranx ruber*, *Lutjanus synagris* and *Sphyraena barracuda* (Fig 7B), while the snapper *Lutjanus synagris* also had high relative abundance on mesophotic reefs (Fig 4). This pattern could in part be explained by factors such as behavioural differences in response to divers [27], fish identification challenges in low light environments [13], or habitat heterogeneity at mesophotic depths [25].

Despite large bodied carnivores having been shown to avoid divers in fished areas [27], few dives are conducted to mesophotic depths on Utila, and mesophotic fish biomass is known to be retained despite shallow fisheries [18,19]. The increased relative biomass of carnivores could reflect reduced diver avoidance if fishing pressure in this depth range has been limited. Studies have identified that flight initiation distance (FID), the distance at which a fish flees from a diver, can be greater in areas with current or previous fishing [5], and FID is naturally higher for larger individuals in many fish families [48]. However, FID variation with fishing pressure does not appear to occur consistently in all families, having been shown to be present in Scaridae [48,49], but not in Lutjanidae or Serranidae [5].

Another explanation for increased carnivore relative community biomass on DOV is declining light intensity with depth. Identification of small fish using DOV is harder on mesophotic than shallow reefs, while video has also been reported to make identification harder through reduced clarity compared to the human eye [13]. The lack of resolution using video could bias DOV at depth to larger bodied fish that are more readily distinguishable during analysis. With the BRUV system static on the seabed for 50 minutes the likelihood of visually identifying small-bodied species is increased through their movement. Although not used here, artificial lighting has been used to good effect on night BRUV surveys [50], and its use on all BRUV and DOV surveys could help increase detection of small individuals.

Habitat heterogeneity is another factor that might drive observed differences between techniques with depth. Harvey *et al*. [25] reports significant habitat:technique interactions when testing un-baited against baited remote video systems on the Great Barrier Reef. They found no differences between techniques on coarse sand and rubble patches or where the camera system landed a short distance away from more structurally complex benthic habitats. However, they did find differences when surveying fine sand/mud and more complex habitats (reefs and macroalgal, sponge and gorgonian beds). While the shallow reefs surveyed here were continuous spur and groove systems, mesophotic reefs were a large patch reef system with reefs separated by areas of fine sand and mud. By the linear nature of DOV surveys, following a depth contour ensured that transects incorporated both the patch reefs and the areas separating them. This level of habitat heterogeneity presents significant challenges to remotely deployed point count methods such as BRUVs. As the BRUV is deployed from a boat, guiding it onto a reef patch is not easily possible. We used a depth sounder to measure depth, and dropped BRUVs in areas known to have reef from exploratory dive surveys, but it was not possible to ensure that direct reef contact would be made, meaning replicates include non-reef habitats adjacent to reefs. While we would expect overall fish biomass to be lower from BRUVs on non-reef areas, large carnivores are some of the most mobile fish on reefs [51]. Therefore we would expect relative community biomass of carnivores to increase in BRUV data from non-reef areas compared to reef areas, caused by large carnivores swimming off patch reefs attracted by the bait, while other fish trophic groups less readily leave the reef. This suggests that mesophotic BRUVs would be biased to carnivores, however, we found greater relative biomass of carnivores via DOVs.

While herbivorous fish relative community biomass was consistently greater on both shallow and mesophotic reefs when surveyed by DOV than BRUV (S2 Fig), the responses we saw to method and depth were often family specific and varied based on whether weighting the community on biomass or abundance. Acanthuridae and Scaridae were both recorded at higher relative abundances by BRUV than DOV on shallow reefs, with the reverse on mesophotic reefs. This pattern is likely to be caused by the high abundance of Labridae and Pomacentridae on shallow reefs detected by DOV. Scaridae, which has a crucial role in algal grazing on western Atlantic reefs [52], was consistently recorded at greater relative community biomass on DOV than BRUV (Table 2, Table 3). As herbivores are unlikely to be attracted by the bait plume of BRUVs, they are likely to make up a lower proportion of BRUV surveyed community biomass. This effect may be particularly accentuated with depth, as previous studies have shown that herbivorous reef fish biomass generally declines with depth [53], including on Utila based on DOV surveys [44]. This suggests that for studies specifically interested in herbivorous reef fish on mesophotic reefs DOV would be the preferred survey method.

Some of the species level relative biomass variation with depth and between methods might be caused by individuals being at different stages in their life cycle. Many tropical western Atlantic fish species undergo ontogenetic migrations, normally from shallow to deeper marine habitats [28]. Andradi-Brown *et al*. [44] looked at length distributions across the depth gradient in reef fish on Utila, and identified several species occurring at larger body sizes at mesophotic depths than shallow depths. Two of these species were the herbivore *Scarus iserti*, and the planktivore *Thalassoma bifasciatum*, both of which were consistently recorded at greater relative biomass at mesophotic depths on DOV than BRUV (Fig 7), and greater or similar abundances on DOV (Fig 4). A third species, the carnivore *Ocyurus chrysurus*, as previously mentioned, was recorded at increased abundance (Fig 4) and relative community biomass (Fig 7) on the BRUV. While we did not test specifically for individual species length differences between the two depths and methods, our results suggest future studies should look at length distributions generated by the two techniques. As BRUV uses MaxN to assess each species, in species that highly aggregate as juveniles, such as *Thalassoma bifasciatum* [54], MaxN is likely to represent juvenile aggregations generating a biased length distribution for the species despite more mature individuals potentially appearing at different times on the BRUV drop. DOV is likely to provide a more uniform length distribution as all individuals of a species within the transect area are measured. This will be particularly apparent for non-aggregating common planktivorous species such as *Chromis cyanea*, making up >30% of the observed individual fish on shallow reefs by DOV, but not attracted to the BRUV. This is supported by much greater abundance of Labridae and Pomacentridae on DOV than BRUV on shallow reefs, with all other families similar between the two methods or more abundant on BRUV (Fig 3). Studies interested in fish length distributions should therefore consider individual fish species behaviour and ontogeny both on the reef and in response to divers when deciding whether to use BRUV or DOV, as both methods have biases that will likely affect length distributions.

In addition to the differences in recorded fish community between the two techniques, there are other practical constraints to consider when deciding whether to use BRUV or DOV for surveys of shallow or mesophotic reefs. Regardless of the method selection, SVS video analysis is time consuming and labour intensive. Video processing times vary greatly based on fish abundance, resulting in shallow reef surveys typically taking longer than mesophotic surveys to analyse. The three minutes of DOV footage per transect took approximately 30–60 minutes to analyse, while the equivalent 50 minutes of BRUV took approximately 240–300 minutes to analyse. Therefore, despite BRUV capturing extra species (Table 1), there is a trade-off with the extra analysis required. If species richness is of interest, the addition of more DOV replicates might be preferable over BRUV to reduce video analysis time. However, while DOV surveys may be quicker to analyse, conducting mesophotic DOV surveys, requires advanced dive training and equipment, specialist dive safety management and detailed planning [55]. This makes DOV surveys, particularly for the lower mesophotic (reefs from 60–150 m), very costly and logistically challenging to conduct, especially in remote field settings. Therefore, while the comparisons outlined in this paper focus on differences in recorded fish community between techniques, the practical logistics of fieldwork may play a major role in dictating which technique should be used.

## Conclusion

We compared the recorded fish community by diver-operated stereo-video (DOV) and baited remote underwater stereo-video (BRUV) surveys on shallow and mesophotic reefs on Utila, Honduras. We detected differences between both techniques in recorded fish communities, but importantly we found differences between techniques varied with depth. We show these differences affect recorded relative community biomass of different trophic groups, including large carnivorous fish often targeted by fisheries. BRUV recorded greater species richness at both shallow and mesophotic depths, making it most appropriate for recording all components of the fish community. DOV however recorded greater relative community biomass of herbivorous reef fish, suggesting studies interested in herbivores specifically should consider using DOV. It is therefore important for researchers and those designing reef fish monitoring programs that span depth gradients to carefully consider what attributes of the reef fish community they are most interested in surveying, and how these various biases in survey technique will affect the ultimate interpretation of their results.

## Supporting Information

S1 Data Raw data used in analysis. Each row represents a unique fish measured during surveys. Columns contain ‘method’ (BRUV or DOV), ‘depth’ (5 or 40, representing shallow or mesophotic respectively), ‘name’ (fish name in the format Family_Genus_species), ‘weight.g’ (estimated fish weight in g), ‘length.mm’ (fish length in mm), ‘family’ (fish family), ‘genus’ (fish genus), ‘species’ (fish species), ‘opcode’ (details of when the survey was conducted in format Site_Depth_Day_Month_Year), ‘site’ (survey site, STP—Stingray Point, LBI—Little Bight, BCW—Black Coral Wall and LHO—Lighthouse reef) and ‘replicate’ (the transect/BRUV drop replicate number at that site and depth).
(CSV)

S1 Fig **Fish relative biomass community structure principal coordinates analysis plot for each site: (A) Stingray Point, (B) Little Bight, (C) Black Coral Wall and (D) Lighthouse reef.** The proportion of variation explained by each axis is shown in brackets.
(TIFF)

S2 Fig **Differences in relative community biomass recorded by the two survey techniques (BRUV and DOV) at each depth for (A) herbivores and (B) carnivores.** Mean ± 1 SE shown, p values indicate whether differences are significant, calculated by a Euclidian permutational ANOVA between the two methods at each depth (see S5 Table for full permutational ANOVA results).
(TIFF)

S1 Table GPS Coordinates for survey locations. Points listed under the column Depth as ‘Shallow/Mesophotic’ represent GPS coordinates of fixed mooring buoys on the reef crest at the sites. For shallow and mesophotic DOV surveys divers descended from these mooring buoys to the survey depth (5 m or 40 m) and conducted two transects east and two transects west from the mooring line. Transects in both directions were started 10 m along the reef from the indicated GPS point. In addition, shallow BRUV surveys were conducted both east and west of these fixed mooring buoys at 5 m depth spaced at approximately 20 m intervals on the reef crest. Mesophotic BRUV drops were deployed by boat, with GPS coordinates for each drop recorded. Mesophotic replicates are named in the form Site_Depth_Day_Month_Year. All GPS coordinates collected on a Garmin GPS unit and recorded in WGS 84.
(DOCX)

S2 Table Euclidian permutational ANOVA testing differences in species richness recorded by the two methods (DOV and BRUV) across both sites and depths. Permutations were constrained within Site and the model simplified to remove non-significant interactions.
(DOCX)

S3 Table Euclidian permutational ANOVA testing differences in total fish biomass recorded by the two methods (DOV and BRUV) across both sites and depths. Permutations were constrained within Site and the model simplified to remove non-significant interactions.
(DOCX)

S4 Table Permutational MANOVA of Bray-Curtis relative fish species community biomass dissimilarities recorded by the two methods (DOV and BRUV) across both sites and depths. Permutations were constrained within Site and the model simplified to remove non-significant interactions.
(DOCX)

S5 Table Full statistical analysis results for S2 Fig. Euclidian permutational ANOVA testing differences in relative fish biomass recorded by the two methods (DOV and BRUV) for (A) shallow herbivores, (B) mesophotic herbivores, (C) shallow carnivores and (D) mesophotic carnivores.
(DOCX)
