# Supplementary figures and images for: Assessing Caribbean Shallow and Mesophotic Reef Fish Communities Using Baited-Remote Underwater Video (BRUV) and Diver-Operated Video (DOV) Survey Techniques
Source: PLoS One. 2016 Dec 13;11(12):e0168235. doi: 10.1371/journal.pone.0168235 (PMC5154558; doi:10.1371/journal.pone.0168235)

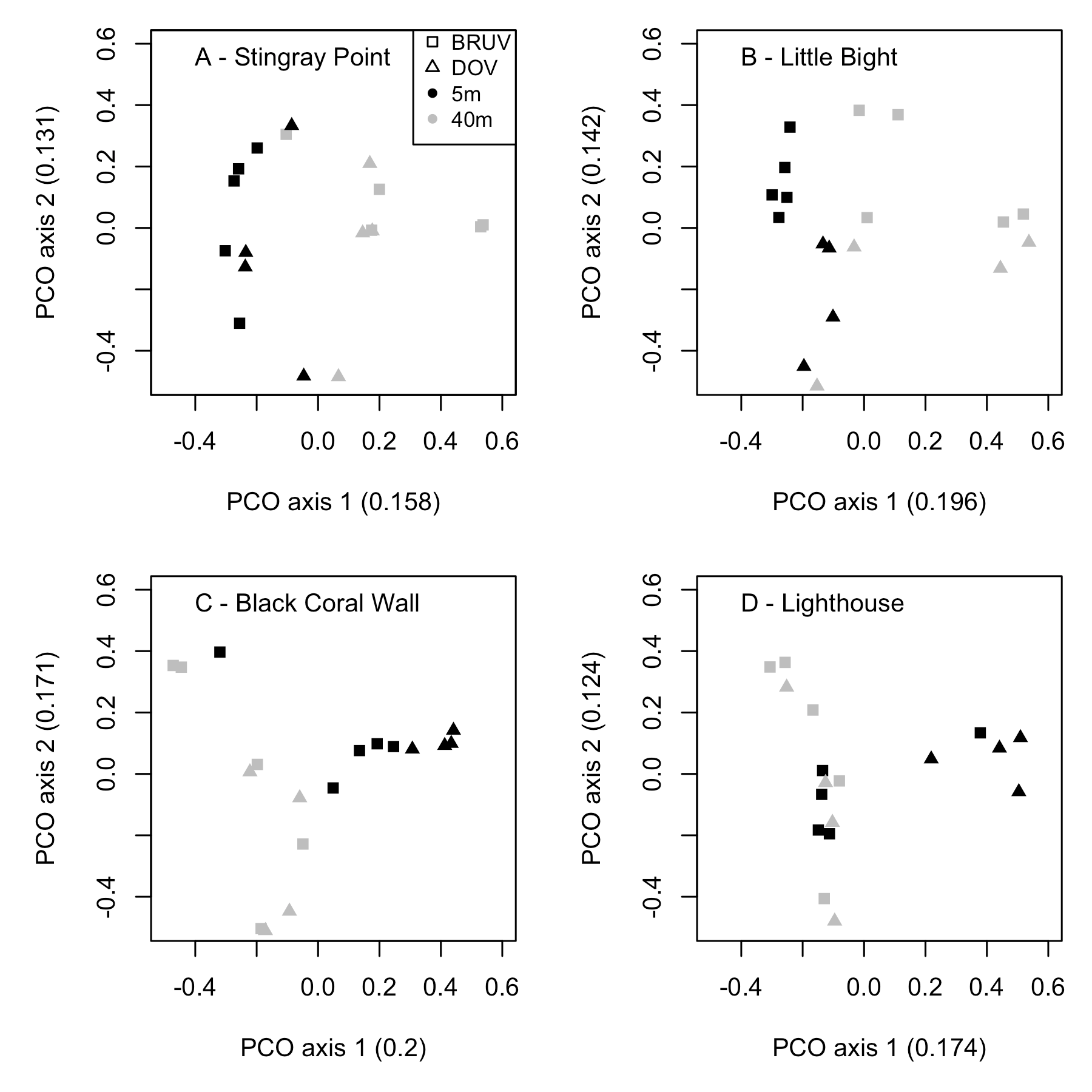

Supplement: S1 Fig — Fish relative biomass community structure principal coordinates analysis plot for each site: (A) Stingray Point, (B) Little Bight, (C) Black Coral Wall and (D) Lighthouse reef. The proportion of variation explained by each axis is shown in brackets. (TIFF) [file pone.0168235.s002.tiff]

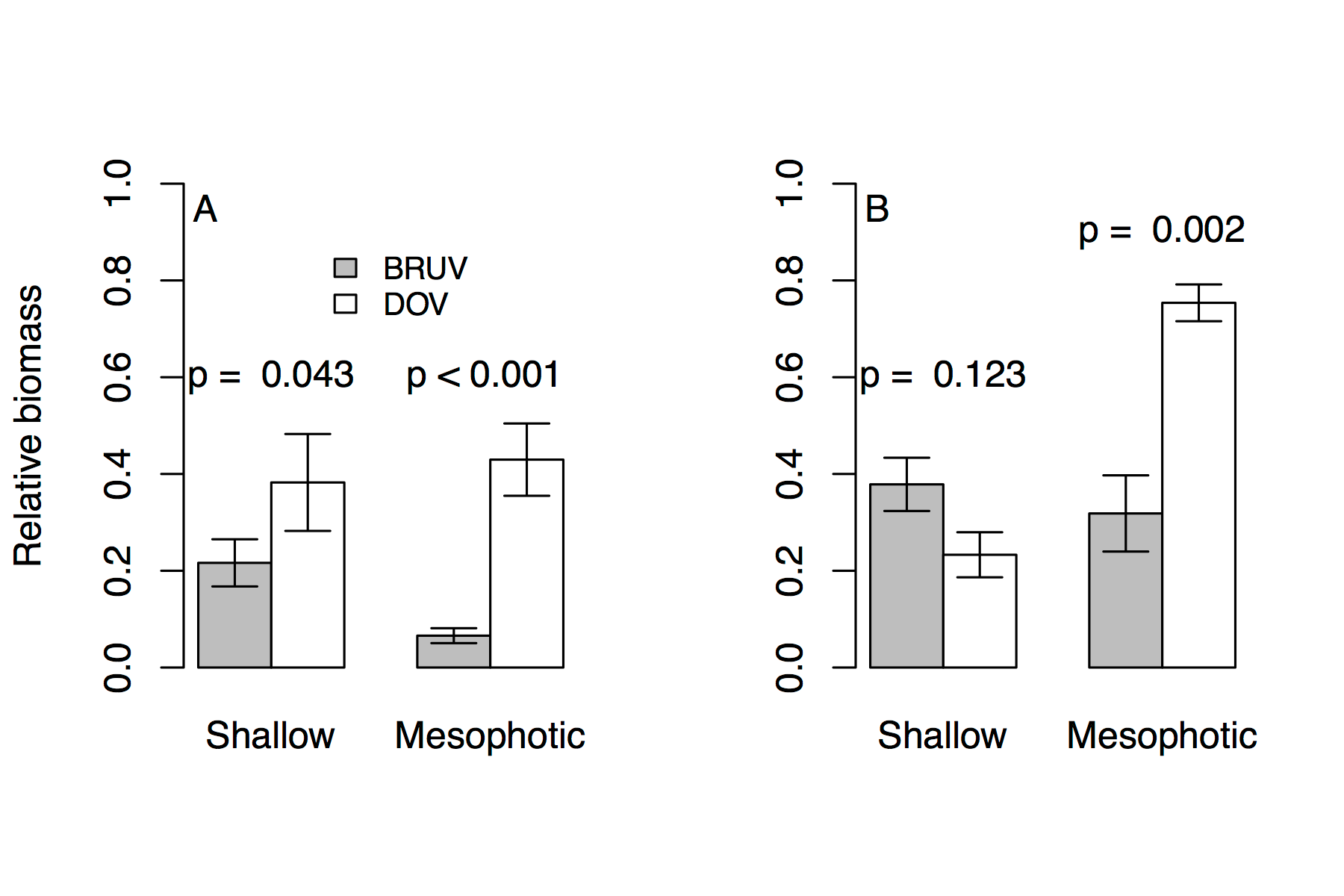

Supplement: S2 Fig — Differences in relative community biomass recorded by the two survey techniques (BRUV and DOV) at each depth for (A) herbivores and (B) carnivores. Mean ± 1 SE shown, p values indicate whether differences are significant, calculated by a Euclidian permutational ANOVA between the two methods at each depth (see S5 Table for full permutational ANOVA results). (TIFF) [file pone.0168235.s003.tiff]
