# Supplementary material for: Assessing Caribbean Shallow and Mesophotic Reef Fish Communities Using Baited-Remote Underwater Video (BRUV) and Diver-Operated Video (DOV) Survey Techniques
Source: PLoS One. 2016 Dec 13;11(12):e0168235. doi: 10.1371/journal.pone.0168235 (PMC5154558; doi:10.1371/journal.pone.0168235)
Supplement: S1 Table — Points listed under the column Depth as ‘Shallow/Mesophotic’ represent GPS coordinates of fixed mooring buoys on the reef crest at the sites. For shallow and mesophotic DOV surveys divers descended from these mooring buoys to the survey depth (5 m or 40 m) and conducted two transects east and two transects west from the mooring line. Transects in both directions were started 10 m along the reef from the indicated GPS point. In addition, shallow BRUV surveys were conducted both east and west of these fixed mooring buoys at 5 m depth spaced at approximately 20 m intervals on the reef crest. Mesophotic BRUV drops were deployed by boat, with GPS coordinates for each drop recorded. Mesophotic replicates are named in the form Site_Depth_Day_Month_Year. All GPS coordinates collected on a Garmin GPS unit and recorded in WGS 84. (DOCX) [file pone.0168235.s004.docx]

**S1 Table. GPS Coordinates for survey locations.** Points listed under the column Depth as ‘Shallow/Mesophotic’ represent GPS coordinates of fixed mooring buoys on the reef crest at the sites. For shallow and mesophotic DOV surveys divers descended from these mooring buoys to the survey depth (5 m or 40 m) and conducted two transects east and two transects west from the mooring line. Transects in both directions were started 10 m along the reef from the indicated GPS point. In addition, shallow BRUV surveys were conducted both east and west of these fixed mooring buoys at 5 m depth spaced at approximately 20 m intervals on the reef crest. Mesophotic BRUV drops were deployed by boat, with GPS coordinates for each drop recorded. Mesophotic replicates are named in the form Site_Depth_Day_Month_Year. All GPS coordinates collected on a Garmin GPS unit and recorded in WGS 84.

| Depth | Site | Replicate | Latitude | Longitude |
| --- | --- | --- | --- | --- |
| Shallow/ Mesophotic | Stingray Point | Shallow DOV and BRUV and Mesophotic DOV | 16.0689055 | -86.95477948 |
| Shallow/ Mesophotic | Little Bight | Shallow DOV and BRUV and Mesophotic DOV | 16.07926302 | -86.92942222 |
| Shallow/ Mesophotic | Black Coral Wall | Shallow DOV and BRUV and Mesophotic DOV | 16.08305968 | -86.91699554 |
| Shallow/ Mesophotic | Lighthouse Reef | Shallow DOV and BRUV and Mesophotic DOV | 16.08534778 | -86.89803786 |
| Mesophotic | Stingray Point | SR_45m_07_08_14 | 16.069732 | -86.95252 |
| Mesophotic | Stingray Point | SR_55m_11_08_14 | 16.067316 | -86.952449 |
| Mesophotic | Stingray Point | SR_50m_11_08_14 | 16.069242 | -86.95097 |
| Mesophotic | Stingray Point | SR_45m_09_07_14 | 16.067022 | -86.955091 |
| Mesophotic | Stingray Point | SR_40m_11_07_14 | 16.065148 | -86.95621 |
| Mesophotic | Little Bight | LB_40m_01_08_14 | 16.074428 | -86.92763 |
| Mesophotic | Little Bight | LB_50m_18_07_14 | 16.075742 | -86.926418 |
| Mesophotic | Little Bight | LB_50m_13_07_14 | 16.075578 | -86.928331 |
| Mesophotic | Little Bight | LB_55m_28_06_14 | 16.075331 | -86.929724 |
| Mesophotic | Little Bight | LB_49m_27_06_14 | 16.077433 | -86.929066 |
| Mesophotic | Black Coral Wall | BCW_45m_28_06_14 | 16.082718 | -86.917268 |
| Mesophotic | Black Coral Wall | BCW_44m_27_06_14 | 16.083609 | -86.915567 |
| Mesophotic | Black Coral Wall | BCW_45m_11_07_14 | 16.082366 | -86.917158 |
| Mesophotic | Black Coral Wall | BCW_40m_31_07_14 | 16.080342 | -86.915708 |
| Mesophotic | Black Coral Wall | BCW_50m_12_07_14 | 16.081651 | -86.916106 |
| Mesophotic | Lighthouse Reef | LH_30m_11_08_14 | 16.079121 | -86.902081 |
| Mesophotic | Lighthouse Reef | LH_45m_09_08_14 | 16.079937 | -86.897165 |
| Mesophotic | Lighthouse Reef | LH_45m_09_08_14 | 16.079163 | -86.900483 |
| Mesophotic | Lighthouse Reef | LH_30m_19_07_14 | 16.08375 | -86.89688 |
| Mesophotic | Lighthouse Reef | LH_30m_13_07_14 | 16.083395 | -86.898666 |
