# Supplementary material for: Assessing Caribbean Shallow and Mesophotic Reef Fish Communities Using Baited-Remote Underwater Video (BRUV) and Diver-Operated Video (DOV) Survey Techniques
Source: PLoS One. 2016 Dec 13;11(12):e0168235. doi: 10.1371/journal.pone.0168235 (PMC5154558; doi:10.1371/journal.pone.0168235)
Supplement: S2 Table — Permutations were constrained within Site and the model simplified to remove non-significant interactions. (DOCX) [file pone.0168235.s005.docx]

**S2 Table. Euclidian permutational ANOVA testing differences in species richness recorded by the two methods (DOV and BRUV) across both sites and depths.** Permutations were constrained within Site and the model simplified to remove non-significant interactions.

| Source | df | MS | F | *p* |
| --- | --- | --- | --- | --- |
| Site | 3 | 36.70 | 2.15 | <0.001 |
| Depth | 1 | 2713.39 | 158.85 | <0.001 |
| Method | 1 | 1915.92 | 112.16 | <0.001 |
| Site:Method | 3 | 54.17 | 3.17 | 0.034 |
| Depth:Method | 1 | 430.12 | 25.18 | <0.001 |
| Residuals | 62 | 17.08 |  |  |
| Total | 71 |  |  |  |
