# Supplementary material for: Assessing Caribbean Shallow and Mesophotic Reef Fish Communities Using Baited-Remote Underwater Video (BRUV) and Diver-Operated Video (DOV) Survey Techniques
Source: PLoS One. 2016 Dec 13;11(12):e0168235. doi: 10.1371/journal.pone.0168235 (PMC5154558; doi:10.1371/journal.pone.0168235)
Supplement: S3 Table — Permutations were constrained within Site and the model simplified to remove non-significant interactions. (DOCX) [file pone.0168235.s006.docx]

**S3 Table. Euclidian permutational ANOVA** **testing differences in total fish biomass recorded by the two methods (DOV and BRUV) across both sites and depths.** Permutations were constrained within Site and the model simplified to remove non-significant interactions.

| Source | df | MS | F | *p* |
| --- | --- | --- | --- | --- |
| Site | 3 | 1364.80 | 2.58 | <0.001 |
| Depth | 1 | 2591.40 | 4.89 | 0.026 |
| Method | 1 | 7233.70 | 13.66 | <0.001 |
| Site:Depth | 3 | 1721.20 | 3.25 | 0.027 |
| Residuals | 63 | 529.40 |  |  |
| Total | 71 |  |  |  |
