# Supplementary material for: Assessing Caribbean Shallow and Mesophotic Reef Fish Communities Using Baited-Remote Underwater Video (BRUV) and Diver-Operated Video (DOV) Survey Techniques
Source: PLoS One. 2016 Dec 13;11(12):e0168235. doi: 10.1371/journal.pone.0168235 (PMC5154558; doi:10.1371/journal.pone.0168235)
Supplement: S4 Table — Permutations were constrained within Site and the model simplified to remove non-significant interactions. (DOCX) [file pone.0168235.s007.docx]

**S4 Table. Permutational MANOVA** **of Bray-Curtis relative fish species community biomass dissimilarities recorded by the two methods (DOV and BRUV) across both sites and depths.** Permutations were constrained within Site and the model simplified to remove non-significant interactions.

| Source | df | MS | F | *p* |
| --- | --- | --- | --- | --- |
| Site | 3 | 0.53 | 1.44 | <0.001 |
| Depth | 1 | 2.32 | 6.28 | <0.001 |
| Method | 1 | 1.75 | 4.74 | <0.001 |
| Site:Method | 3 | 0.56 | 1.53 | 0.004 |
| Depth:Method | 1 | 0.91 | 2.47 | <0.001 |
| Residuals | 62 | 0.37 |  |  |
| Total | 71 |  |  |  |
