# Supplementary material for: Assessing Caribbean Shallow and Mesophotic Reef Fish Communities Using Baited-Remote Underwater Video (BRUV) and Diver-Operated Video (DOV) Survey Techniques
Source: PLoS One. 2016 Dec 13;11(12):e0168235. doi: 10.1371/journal.pone.0168235 (PMC5154558; doi:10.1371/journal.pone.0168235)
Supplement: S5 Table — Euclidian permutational ANOVA testing differences in relative fish biomass recorded by the two methods (DOV and BRUV) for (A) shallow herbivores, (B) mesophotic herbivores, (C) shallow carnivores and (D) mesophotic carnivores. (DOCX) [file pone.0168235.s008.docx]

**S5 Table. Full statistical analysis results for S2 Fig.** Euclidian permutational ANOVA testing differences in relative fish biomass recorded by the two methods (DOV and BRUV) for (A) shallow herbivores, (B) mesophotic herbivores, (C) shallow carnivores and (D) mesophotic carnivores.

A. Shallow Herbivores:

| Source | df | MS | F | *p* |
| --- | --- | --- | --- | --- |
| Method | 1 | 0.25 | 4.18 | 0.041 |
| Residuals | 34 | 0.06 |  |  |
| Total | 35 |  |  |  |

B. Deep herbivores:

| Source | df | MS | F | *p* |
| --- | --- | --- | --- | --- |
| Method | 1 | 0.42 | 8.21 | 0.006 |
| Residuals | 12 | 0.05 |  |  |
| Total | 13 |  |  |  |

C. Shallow Carnivores:

| Source | df | MS | F | *p* |
| --- | --- | --- | --- | --- |
| Method | 1 | 0.13 | 2.59 | 0.129 |
| Residuals | 31 | 0.05 |  |  |
| Total | 32 |  |  |  |

D. Mesophotic Carnivores:

| Source | df | MS | F | *p* |
| --- | --- | --- | --- | --- |
| Method | 1 | 1.08 | 14.02 | <0.001 |
| Residuals | 25 | 0.08 |  |  |
| Total | 26 |  |  |  |

| \|  \| \| --- \| |
| --- | --- |
